# Role of hypermethylated SLC5A8 in follicular thyroid cancer diagnosis and prognosis prediction

**DOI:** 10.1186/s12957-023-03240-1

**Published:** 2023-11-25

**Authors:** Yan Yang, Chenjin Liao, Qian Yang, Yuxia Li, Yunxiang Tang, Bin Xu

**Affiliations:** 1 Department of Oncology and Hematology, Chongqing University Central Hospital, Chongqing, 400014 China; 2 Department of Ultrasound, Chongqing University Central Hospital, Chongqing, 400014 China; 3 Department of General Surgery, Chongqing University Central Hospital, Chongqing, 400014 China; 4 Department of Nuclear Medicine, Chongqing University Central Hospital, Chongqing, 400014 China

**Keywords:** SLC5A8, Thyroid cancer, Follicular, DNA, mRNA

## Abstract

**Objective:**

Thyroid cancer is one of the most frequently reported endocrine system malignancies. It is difficult to distinguish follicular thyroid cancer (FTC) from follicular thyroid adenoma (FTA) during pathological diagnosis in patients without lymph nodes or distant metastases. Therefore, we conducted a retrospective study to investigate the significance of SLC5A8 methylation and expression in the diagnosis and prognosis of FTC.

**Methods:**

We used 165 tissue samples, including FTC (*n* = 58), thyroid tumors of uncertain malignant potential (TT-UMP, *n* = 40), and FTA (*n* = 67), to explore the differences in SLC5A8 methylation and mRNA transcription in different pathological types. Survival analysis was conducted to evaluate the recurrence rate at a 5-year follow-up.

**Results:**

The SLC5A8 methylation positive rate was higher in patients with thyroglobulin ≥ 40 μg/l and Chol ≥ 5.17 mmol/l, and it was higher in patients with FTC (*n* = 42, 72.4%) than those with FTA (*n* = 27, 40.3%) and TT-UMP (*n* = 23, 57.5%). The relative concentration of SLC5A8 mRNA was lower in patients with FTC than in those with FTA (*p* < 0.05). At 5-year follow-ups, patients who were SLC5A8 methylation-positive had a higher recurrence rate than those who were methylation-negative.

**Conclusions:**

Our current study indicates that SLC5A8 gene methylation occurs more commonly in patients with FTC than in those with FTA. The differences in SLC5A8 methylation and expression among FTA, FTC, and TT-UMP provide an important basis for further exploration of epigenetic changes in the occurrence, development, and prognosis of thyroid cancer. Our findings need to be further validated in larger populations with long-term follow-up in the future.

**Supplementary Information:**

The online version contains supplementary material available at 10.1186/s12957-023-03240-1.

## Introduction

Thyroid cancer is one of the fastest-growing and most frequently reported malignancies of the endocrine system [1, 2]. Its incidence has rapidly increased over the past decade, occurring at a rate of 6.7/100,000 person-years in 2018. It is characterized by high genetic heterogeneity, disease aggressiveness, and various pathological entities [3–5]. Differentiated thyroid cancer (DTC) accounts for more than 90% of all new-onset thyroid cancers and has received increasing attention in recent years [6]. DTC includes papillary thyroid cancers (PTC), follicular thyroid cancers (FTC), and oncocytic cell thyroid cancers [7]. However, the histological manifestations of FTC and follicular thyroid adenoma (FTA) are similar, thus creating a problem with the molecular diagnosis of DTC. Distinguishing FTC from FTA in the pathological diagnosis of patients without lymph nodes or distant metastases is difficult [8]. However, if diagnosed at an early stage, many treatment options are available for patients with malignant thyroid cancer and they usually have a promising prognosis [9]. However, Grønlund and colleagues reported that approximately 11–39% of patients with FTC develop cancer recurrence [10]. Thus, there is an urgent need to improve the accuracy of DTC diagnosis and identify the risk of cancer recurrence and metastasis.

With the development of next-generation sequencing and protein mass spectrometry, accumulating evidence has revealed differences in gene mutation sites, mRNA, and protein expression between FTC and FTA [11]. The SLC5A8 gene was first discovered in the thyroid in 2002 in 12Q13-23 of the human chromosome [12]. Previous studies have shown that SLC5A8 is predominantly found in the small intestine, colon, kidney, thyroid, and salivary glands and, to a lesser extent, in the retina and brain [13, 14]. Accumulating evidence has confirmed that SLC5A8 exerts proliferative effects on tumor-dearing and anti-tumor cell proliferation [15]. The methylation status of SLC5A8 has been reported in various cancers, including thyroid, esophageal, and breast cancer [16–18]. Detection of SLC5A8 methylation has also been reported, which may contribute to the early diagnosis and prognosis of thyroid cancers [19].

To date, only a few studies have reported the role of SLC5A8 methylation in the early diagnosis of benign and malignant thyroid cancers. However, the specific features of SLC5A8 methylation between FTC and FTA and whether SLC5A8 methylation contributes to the prediction of FTC prognosis have not been fully studied. Moreover, thyroid tumors of uncertain malignant potential (TT-UMP) refer to the presence of questionable capsular/vascular invasion or incompletely developed PTC-type nuclear changes [20, 21]. These tumors are also difficult to diagnose in most cases. Thus, a great challenge in differentiating between FTC, FTA, and TT-UMP is the difference between observers in the histologic interpretation of capsular or vascular invasion, which largely depends on the pathologists, even in surgically resected samples [22]. We conducted a novel retrospective study to investigate the significance of SLC5A8 methylation in the diagnosis and prognosis of FTC.

## Subjects and methods

### Ethical approval

The Research Ethics Committee of Chongqing University Central Hospital reviewed and approved the study protocols (IRB2018032). All participants provided written informed consent prior to inclusion, and all procedures were performed in accordance with the 2013 revised Declaration of Helsinki.

### Study population

To detect SLC5A8 methylation, 165 tissue samples were collected immediately after the resection surgery, including FTC (*n* = 58), TT-UMP (*n* = 40), and FTA samples (*n* = 67). Eligible patients were enrolled in this study between January 2018 and September 2019 at the Chongqing University Central Hospital, China. The inclusion criteria were as follows: (1) all enrolled patients had clear surgical indications; (2) patients had complete clinical data, laboratory test results, and pathological diagnosis data; (3) no history of clinical treatment for thyroid-related diseases or any anticancer treatment, such as radiotherapy and chemotherapy, before receiving surgical treatment; (4) not diagnosed with any other malignant cancers or genetic diseases; and (5) within the age range of 20–70 years old. The exclusion criteria included patients who did not meet the above inclusion criteria, those with other thyroid diseases, or any other severe or unstable medical illness.

### Outcome measurement and follow-up

We collected demographic and physiological information of all participants, including age, sex, and smoking and drinking habits, through a chart review. A Case Report Form (CRF) was used to collect the clinical data of the patients with FTC, including the number and size of nodules, invasion of the capsule, thyroid-binding globulin (TG), cholesterol, triglyceride, preoperative thyroid function, B-ultrasound characteristics, lymph node metastasis, TNM grading, and other information.

All patients with FTC underwent radioactive iodine (RAI, Supplemental Fig. S2) in the clinical practice. Patients were followed up by reviewing outpatient medical records, inpatient cases, and telephone calls. The biochemical and instrumental investigations took place once every 3 months in the first year; once every 6 months in the second year; once a year in the 3rd to 5th year. The follow-up period was from the postoperative period until September 2021, with a follow-up duration of 5 years. Recurrence of FTC indicates that residual tumors or new lesions are found during follow-up and clinical diagnosis. The last follow-up date and disease status of the patients (no events, persistent disease, recurrence, or disease-specific death) were recorded, along with the type of recurrence (local recurrence, regional recurrence, or distant recurrence) and recurrence date.

### DNA extraction and quantification

#### Primer design

Premier 5.0 software was used to design the SLC5A8 gene primers. The primers were designed by referring to GenBank and synthesized by Shanghai Sanko Co., Ltd. Primer sequences are listed in Table 1. According to the DNA sequence of the SLC5A8 gene, two pairs of primers were designed for the PCR reaction.

**Table 1:** Primer sequences and amplified fragments

Primer	Forward primer	Reverse primer	Length
SCL5A8	5′-ATCTACGGTGTCAACCAATCCC-3′	5′-GCGAGCCCACAAAACACTGAG-3′	134 bp
SCL5A8 unmethylation	5′TTGAATGTATTTTGAGGTG-3′	5′-TCAATTTTCCAAAATCCC-3′	100 bp
SCL5A8 methylation	5′-TCGAACGTATTTCGAGGC-3′	5′-ACAACGAATCGATTTTCCG-3′	108 bp
β-actin	5′-GTGGACATCCGCAAAGAC-3′	5′-AAAGGGTGTAACGCAACTAA-3′	302 bp

#### SLC5A8 gene methylation detection

Methylation-specific polymerase chain reaction (PCR) was performed to detect SLC5A8 methylation. Genomic DNA was extracted from thyroid nodule tissue samples using a tissue genomic DNA extraction kit (Tiangen, Beijing, China), and the bisulfite-modified DNA of the corresponding primers was amplified by PCR. Genomic DNA was treated with sodium bisulfite, purified using the EZ DNA methylation-Gold Kit 3 (Zymo Research, CA, USA), and stored at − 20 °C till further use. Methylation-specific PCR (MSP) was performed as described previously. Specific primers were designed to amplify SLC5A8 genes (Table 1) and were commercially synthesized by TIANGEN (CN). PCR conditions for SLC5A8 were as follows: the PCR reaction conditions were as follows: 50 °C for 2 min; pre-denaturation at 95 °C for 2 min; followed by 40 cycles at 95 °C for 15 s and 60 °C for 30 s. Reaction products were photographed by agarose gel electrophoresis.

#### SLC5A8 gene transcription detection

SLC5A8 (gene) RNA transcription was determined using real-time polymerase chain reaction (RT-PCR). Total RNA was extracted from sample tissues according to the manufacturer’s instructions, followed by reverse transcription. Specifically, total RNA was extracted from tissue samples using the RNAprep pure tissue kit (DP431; Tiangen, Beijing, China) following the manufacturer’s instructions. Purified RNA was stored at – 80 °C till further use. RT-PCR was performed to detect primer specificity. cDNA was synthesized from purified RNA using the First Strand cDNA Synthesis Kit (Catalog number: K1612; Thermo Fisher Scientific) according to the manufacturer’s instructions. The primer sequences used for the SLC5A8 were those described in Table 1 and were commercially synthesized by Invitrogen (USA). PCR reaction conditions were as follows: 50 °C for 2 min; pre-denaturation at 95 °C for 2 min; followed by 40 cycles of 95 °C for 15 s and 60 °C for 30 s; the reaction products were photographed by agarose gel electrophoresis.

#### SLC5A8 gene expression detection

Tissues were lysed, and protein was extracted using the WIP kit (Tissue and Cell lysis solution for Western blot and Immunoprecipitation; Boaoseng Biotechnology, Beijing, China). A bicinchoninic acid assay (Biomed, Beijing, China) was used to quantify total protein concentration. The proteins (60–100 µg) were separated by electrophoresis on a 12% sodium dodecyl sulfate–polyacrylamide gel, transferred to polyvinylidene fluoride (PVDF) membrane, blocked with 10% skimmed milk powder at room temperature (21–25 ℃) for 1 h, and were subsequently incubated with primary antibodies-polyclonal rabbit anti-human SLC5A8 (1:500 dilution; Abcam, ab137214, Shanghai, China) and anti-GADPH (1:500; Beyotime, Beijing, China), overnight at 4 °C. Membranes were washed with TBST buffer and then incubated with a Goat Anti-Rabbit secondary antibody (1:7500 dilution; Abcam ab205718, Shanghai, China) at room temperature (21–25 ℃) for 1.5 h. The blots were visualized using the ECL system (Biyun Days Biotechnology Co., Beijing, China). The relative expression of SLC5A8 protein was determined based on the ratio of the optical density of SLC5A8 to that of GADPH using Gel Pro32 analyzer software.

### Statistical analysis

Descriptive statistics were performed with continuous variables estimated as the mean and standard deviation (SD), and categorical variables were summarized as frequencies and proportions. Chi-square tests were used to test for statistically significant differences in categorical variables between various groups, whereas one-way ANOVA was used to test for differences in continuous variables. Post-hoc analyses for pairwise significance between the two groups were performed using the least significant difference (LSD) method. The Wilcoxon test was used to analyze paired data. The survival curve was drawn according to the Kaplan–Meier method to evaluate the recurrence rate of patients 2 years after surgery. The log-rank test was used for statistical comparison, and the COX proportional hazards regression model was used to evaluate the recurrence risk. *P* values < 0.05 were considered statistically significant. All statistical analyses were performed using SPSS 20.0 software.

## Results

### Demographic characteristics of participants

Among the 165 patients included in the study, the thyroid disease histotypes were as follows: 58 FTC, 67 FTA, and 40 TT-UMP. The mean ages (SD) of patients with FTC, FTA, and TT-UMP were 43.78 ± 13.66, 44.36 ± 15.07, and 43.05 ± 16.27 years old, respectively. The distributions of age, sex, smoking and drinking habits, and tumor size were similar among patients, and the differences were not statistically significant (all *p* > 0.05). Regarding the FTC histologic type, 28 samples (48.28%) were minimally invasive, 16 (27.59%) were encapsulated angioinvasive, and 22 (30.1%) were widely invasive. The basic characteristics of the participants are listed in Table 2.

**Table 2:** The baseline characteristics of included participants

Variables	FTC (*n* = 58)	FTA (*n* = 67)	TT-UMP (*n* = 40)	*P*
Age, year, mean ± SD	43.78 ± 13.66	44.36 ± 15.07	43.05 ± 16.27	0.907
Sex (*n*, %)				
Male	30(51.72)	40(59.7)	21(52.5)	0.621
Female	28(48.28)	27(40.3)	19(47.5)	
Smoker (*n*, %)	9(15.52)	9(13.43)	5(12.5)	0.903
Drinker (*n*, %)	9(15.52)	11(16.42)	6(15)	0.979
Tumor size, cm, Mean ± SD	2.17 ± 1.54	1.91 ± 1.11	1.73 ± 1.08	0.157
Histologic type (*n*, %)				
Minimally invasive	28(48.28)			-
Encapsulated angioinvasive	16 (27.59)			
Widely invasive	14(24.14)			
Disease recurrence (n, %)				
Yes	15(25.86)	–	–	–
No	43(74.14)	67(100)	40(100)	

### Comparison of methylation of the SLC5A8 gene in thyroid cancer tissues with different clinical features

**Fig. 1:**
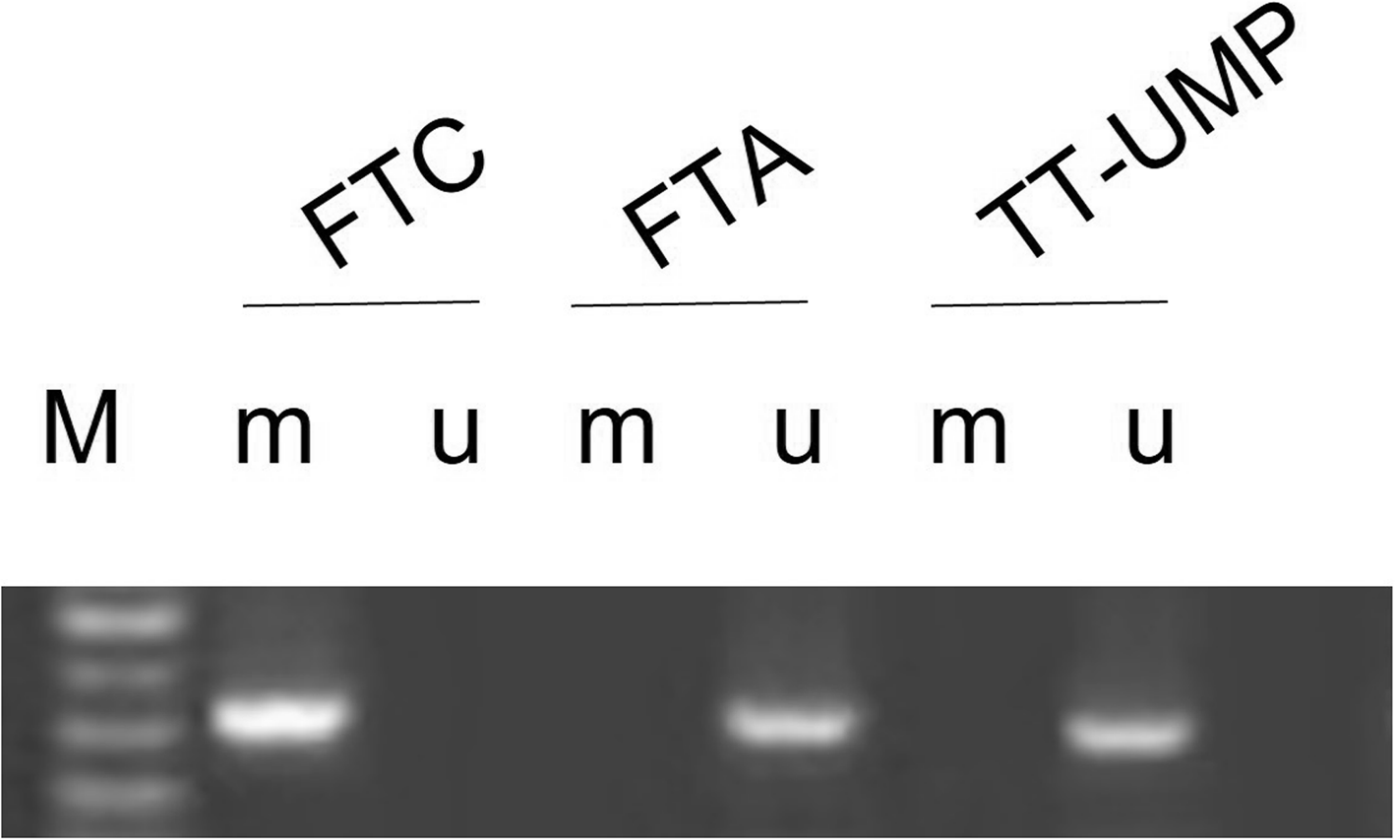
Representative gel images of the SLC5A8 gene methylation-specific PCR in thyroid tissue samples. M: 100 bp marker; m: hypermethylation; u: un-hypermethylation

The comparison of SLC5A8 methylation in thyroid cancer tissues with different clinical features is shown in Table 3 and Supplemental Fig. S1. We found no significant differences in the SLC5A8 methylation positive rate in different groups based on age, sex, tumor size, and triglyceride levels (all *p* > 0.05). The SLC5A8 methylation positive rate was higher in the thyroglobulin ≥ 40 μg/l group when compared with thyroglobulin < 40 μg/l (67.2% vs. 47.6%, *p* = 0.006); the SLC5A8 methylation positive rate was also higher in the Chol ≥ 5.17 mmol/l group when compared with Chol < 5.17 mmol/l group (68.6% vs. 50.0%, *p* = 0.026). For patients with different pathological types, the results of SLC5A8 gene methylation PCR were as follows: methylation amplification product bands were found in 42 (72.4%) tissue samples of FTC; methylation in the FTA group was found in 27 cases, accounting for 40.3%; and 23 cases in the TT-UMP group reported SLC5A8 gene methylation, accounting for 57.5%. There was a statistically significant difference in the positive rate of SLC5A8 methylation among the three groups (*p* = 0.001). Methylation of the SLC5A8 promoter region in thyroid tissues of FTC, FTA, and TT-UMP is shown in Fig. 1. We did not find a significant difference in the methylation of SLC5A8 among the three histological types of patients with FTC (*p* = 0.576); the details are shown in Supplemental Table S1.

**Table 3:** Comparison of SLC5A8 gene methylation in patients with different clinical features in baseline

Variables	*n*	Positive (*n* = 92)	Negative (*n* = 73)	*χ*^2^	*P*
Age					
< 45	110	59(53.6)	51(46.4)	0.602	0.438
≥ 45	55	33(60.0)	22(40.0)		
Sex					
Male	74	42(56.8)	32(43.2)	0.054	0.816
Female	91	50(54.9)	41(45.1)		
Tumor size					
< 1.0 cm	71	41(57.7)	30(42.3)	0.200	0.655
≥ 1.0 cm	94	51(54.3)	43(45.7)		
TG					
< 40 μg/L	91	42(46.2)	49(53.8)	7.586	0.006
≥ 40 μg/L	74	50(67.6)	24(32.4)		
Chol					
< 5.17 mmol/L	114	57(50.0)	57(50.0)	4.956	0.026
≥ 5.17 mmol/L	51	35(68.6)	16(31.4)		
Triglycerides					
< 1.7 mmol/L	134	73(54.5)	61(45.5)	0.474	0.491
≥ 1.7 mmol/L	31	19(61.3)	12(38.7)		
Pathological type					
FTC	58	42(72.4)	16 (27.6)	13.063	0.001
FTA	67	27(40.3)	40 (59.7)		
TT-UMP	40	23(57.5)	17 (42.5)		

Positive means SLC5A8 gene hypermethylation; negative means SLC5A8 gene un-hypermethylation. If the sample has both u and m bands, we will delete the sample and not include this data in the result

### Expression patterns of SLC5A8 mRNA and protein in different pathological types

The results of semi-quantitative RT-PCR reported that the differences of SLC5A8 mRNA in the FTC, FTA, and TT-UMP groups were 58.90 ± 22.49, 89.54 ± 44.54, and 73.56 ± 27.38, respectively.

**Fig. 2:**
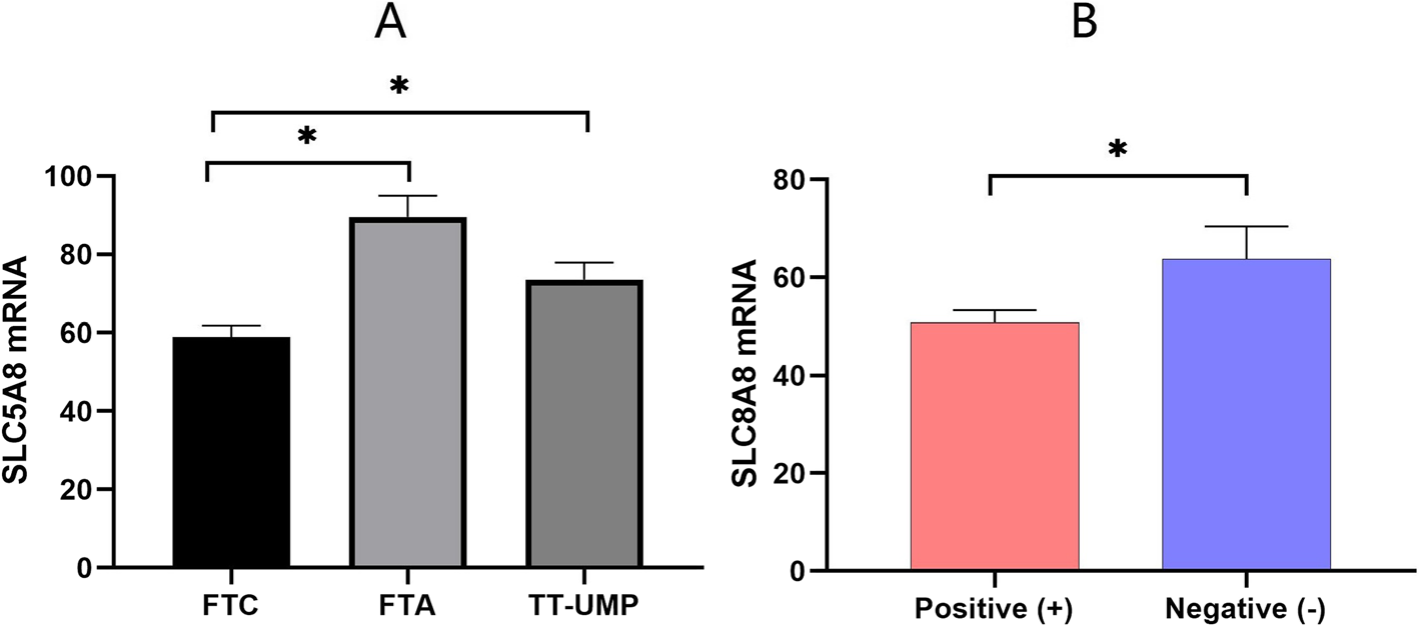
The mRNA transcription level of the SLC5A8 gene in different pathological types. **A** The comparison of mRNA in FTA, FTC, and TT-UMP groups. **B** The comparison of mRNA in patients with positive and negative SLC5A8 gene methylation in the FTC group

**Fig. 3:**
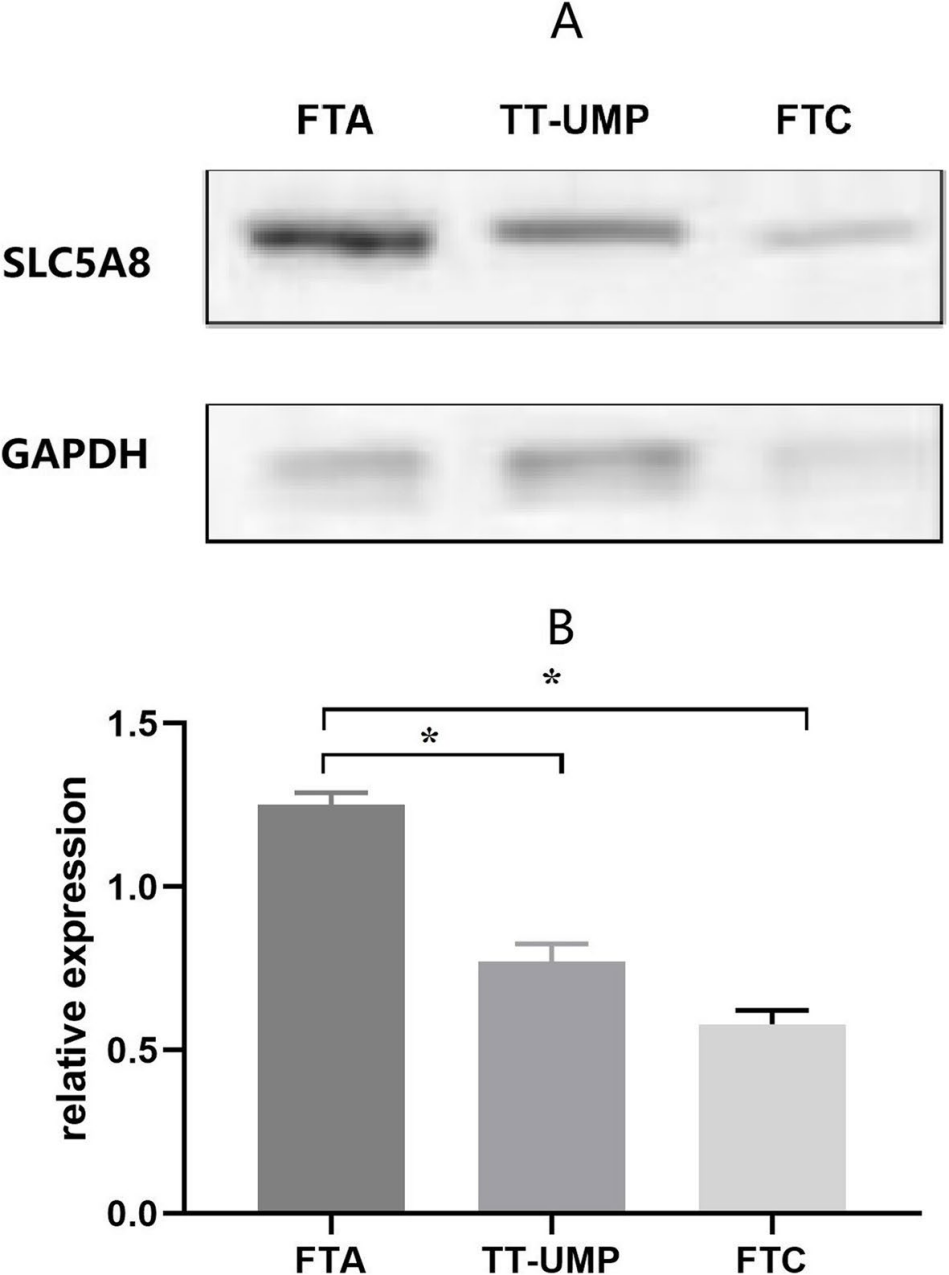
Expression of the SLC5A8 protein in thyroid tissues. **A** Representative immunoblot images of SLC5A8 in FTA, FTC, and TT-UMP groups. **B** Graphical representation of relative protein expression of SLC5A8 in various thyroid tissues. *p < 0.05

We found the ΔCT of SLC5A8 mRNA in the FTC, FTA, and TT-UMP groups was statistically significant (*p* < 0.001). The post-hoc analysis revealed significant differences between the FTC, FTA, and TT-UMP groups. In thyroid tissues, SLC5A8 mRNA expression was higher in FTA and TT-UMP than in FTC. The difference was statistically significant (*p* < 0.05). However, no significant difference was found between the FTC and TT-UMP groups (Fig. 2A). The semi-quantitative RT-PCR results showed that SLC5A8 mRNA in patients with positive and negative SLC5A8 gene methylation in the FTC group were also significantly different (50.76 ± 16.58 vs. 63.79 ± 26.64, *t* = 2.906, *p* = 0.005, Fig. 2B). Post-hoc analysis indicated that SLC5A8 protein levels were significantly lower in FTC tissues than in TT-UMP and FTA tissues (*p* < 0.05, Fig. 3).

### Analysis of disease recurrence in patients with FTC

**Fig. 4:**
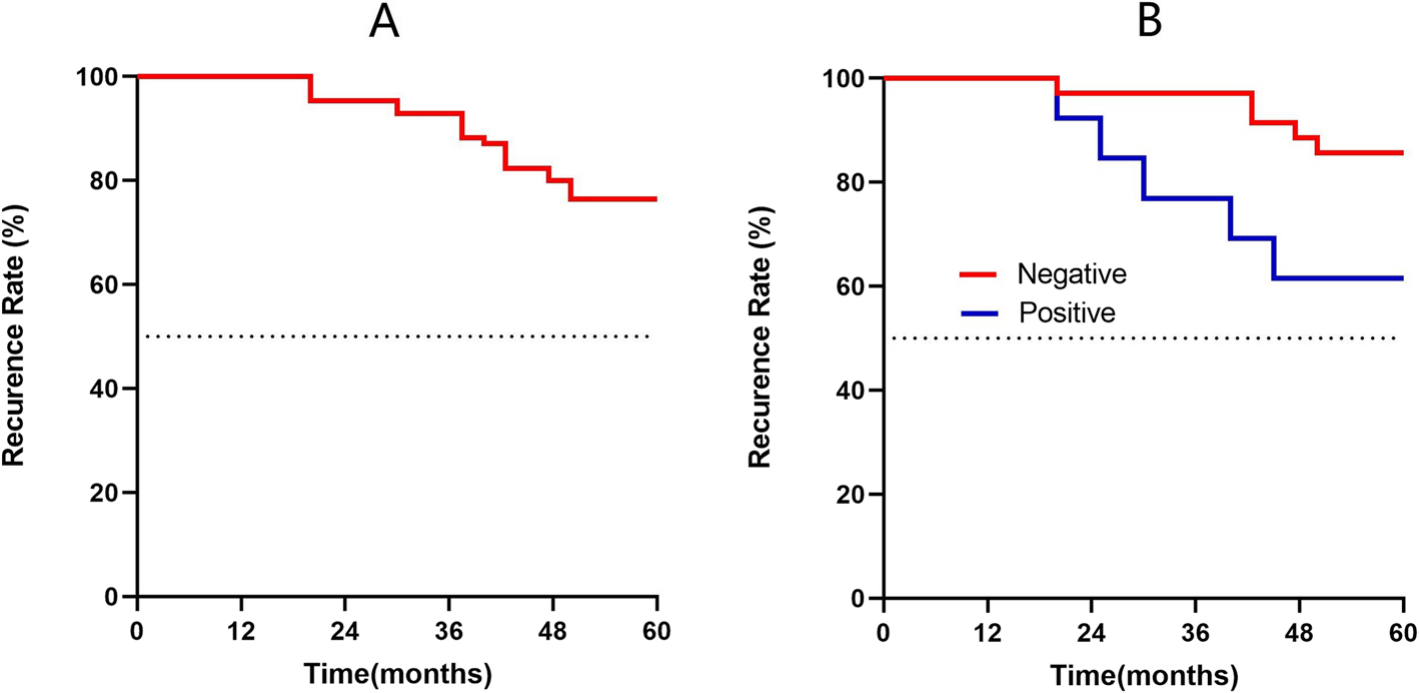
Disease recurrence rate in patients with FTC. **A** Recurrence status in patients with FTC. **B** Comparison of recurrence rate in FTC patients with SLC5A8 gene methylation positive and negative

During the 5-year follow-up, ten patients in the FTC group were lost to follow-up. The recurrence rate in the FTC group was 22.9% (11/48) and the recurrence time ranged from 20–50 months (Fig. 4A). Further analysis showed that the recurrence rate in SLC5A8 methylation-positive patients (5/13, 38.5%) was higher than that in methylation-negative patients (5/35, 14.2%); however, the difference was not statistically significant (*χ*^2^ = 0.4.375, *p* = 0.036) (Fig. 4B).

## Discussion

In this study, we explored the role of SLC5A8 gene methylation and expression in the diagnosis and prognosis of FTC in a Chinese population. We found that (1) the SLC5A8 methylation positive rate was higher in patients with thyroglobulin ≥ 40 μg/l and Chol ≥ 5.17 mmol/l, it was higher in patients with FTC (*n* = 42, 72.4%) than FTA (*n* = 27, 40.3%) and TT-UMP (*n* = 23, 57.5%); (2) the relative concentration of SLC5A8 mRNA was lower in patients with FTC than FTA; (3) the SLC5A8 methylation-positive patients have a higher recurrence rate than negative patients in the 5-year follow-up, which needs to be confirmed in larger samples.

SLC5A8 is a protein-encoding gene. Among the related pathways are the transport of glucose and other sugars, bile salts and organic acids, metal ions, and amine compounds, and metabolism [23]. SLC5A8 also transports monocarboxylates and short-chain fatty acids via a sodium-coupled mechanism [24]. Our study reported that the SLC5A8 methylation positivity rate was associated with high thyroglobulin and cholesterol levels, which may suggest that dysregulation of the SLC5A8 gene is involved in widespread metabolic disturbances in patients with thyroid adenoma and thyroid cancer. However, this result has not been reported in other population-based studies. In contrast to our findings, downregulated SLC5A8 was associated with a lower concentration of glucose and triglyceride and a higher concentration of total cholesterol and low-density lipoprotein cholesterol in plasma in a neonatal piglet study [25]. The difference may be caused by different species and is worth further exploration.

Accumulating evidence has shown that SLC5A8 acts as a tumor suppressor and plays an important role in promoting tumor apoptosis and antitumor cell proliferation [26]. Thus, abnormal DNA methylation of SLC5A8 is considered the standard for determining the specificity of tumor molecules, which has a guiding role in the early diagnosis and prognosis of cancers [18, 27]. Some data from tissue DNA suggest that aberrant methylation of the SLC5A8 gene may be an early change in thyroid tumorigenesis, regardless of the cell type [28]. Our current study reported higher methylation of the SLC5A8 gene in the tissues of patients with FTC. This is consistent with the results of Khatami et al. showing that methylation of the SLC5A8 gene is a common phenomenon in papillary thyroid carcinoma [19]. The detection of SLC5A8 methylation establishes a precise diagnosis and differentiation between FTA and FTC, which helps to compensate for the potential invisibility of routine pathological examinations at the molecular level.

Our study reported lower expression of SLC5A8 mRNA in patients with FTC than in those with FTA and TT-UMP but did not find significant differences between FTA and TT-UMP. Consistent with our finding, down-regulated mRNA expression of SLC5A8 was reported in cervical cancer, hepatocellular carcinoma, and *Helicobacter pylori* infection [16, 29, 30]. Previous evidence suggests that SLC5A8 alleviates disease progression by regulating the *Wnt* signaling pathway [16], which requires further exploration in patients with thyroid cancer.

Moreover, DNA methylation biomarkers have been reported to accurately predict the prognosis of patients with malignant cancers such as colon [31], breast [32], and pancreatic cancers [33]. Our COX multivariate analysis showed that SLC5A8 methylation is one of the risk factors affecting the prognosis of thyroid cancer. The methylation profile of thyroid cancer exhibits a specific signature according to the histological subtype [34], and the predictive potential of DNA methylation, especially of SLC5A8, for thyroid cancer prognosis remains to be further explored. Future research needs to consider completing historical results to better distinguish between FTC and FTA.

This study has several limitations. First, it was conducted in only one hospital in China, and the sample size was relatively small. Particularly, in the follow-up given to the patients, those that were positive for methylation were only 5/13 patients who showed recurrence within a 5-year interval, as described. Therefore, there may have been some degree of bias in the results. Second, we failed to follow up on patients with TT-UMP, there were no deaths in the patients overall during the 5-year follow-up period, and we did not conduct long-term follow-up. Thirdly, we did not conduct further analyses of the other clinical information of the patients at follow-up. In this study, we only measured DNA methylation and mRNA expression of SLC5A8. These results were not been confirmed using DNA methylase inhibitors or immunofluorescence assays. Moreover, we did not include the other two types of DTC patients (i.e., PTC and oncocytic cell thyroid cancers) in the current study. Whether SLC5A8 has the potential to distinguish different DTCs worth further exploration in the future. Finally, the high recurrence rate in the current study may be due to some patients having undergone unilateral thyroidectomy, or because the lost subjects were more likely to have no recurrence. Thus, our findings need to be further validated in larger populations with long-term follow-up periods in the future.

## Conclusions

In summary, our current study indicates that SLC5A8 gene methylation commonly occurs in patients with FTC and its detection can be considered an auxiliary technology for diagnosing and identifying FTC. However, these findings need to be implemented and validated through more experiments and analyses. Moreover, the differences in SLC5A8 gene transcription and expression between FTA, FTC, and TT-UMP provide an important basis for further exploration of epigenetic changes in the occurrence, development, and prognosis of thyroid cancer, and provide possible potential molecular targets for the treatment of these tumors.

### Supplementary Information

**Additional file 1: Supplemental Figure 1.** Comparison of SLC5A8 gene methylation in patients with different clinical features in baseline. **Supplemental Figure 2.** Radioactive iodine (RAI) treatment of representative cases with thyroid cancer metastasis. (A) whole body imaging and (B) computed tomography (CT) of patients who underwent RAI with lung metastasis+lymph node metastasis. (C) whole body imaging and (D) CT of patients who underwent RAI with bone metastasis. **Supplemental Table 1.** The baseline characteristics of included participants.

**Additional file 2.**

## Data Availability

The data that support the findings of this study are available from the corresponding author, [BX], upon reasonable request.
