# Supplementary material for: Role of hypermethylated SLC5A8 in follicular thyroid cancer diagnosis and prognosis prediction
Source: World J Surg Oncol. 2023 Nov 25;21:367. doi: 10.1186/s12957-023-03240-1 (PMC10675931; doi:10.1186/s12957-023-03240-1)
Supplement: Supplementary file 1 — Additional file 1: Supplemental Figure 1. Comparison of SLC5A8 gene methylation in patients with different clinical features in baseline. Supplemental Figure 2. Radioactive iodine (RAI) treatment of representative cases with thyroid cancer metastasis. (A) whole body imaging and (B) computed tomography (CT) of patients who underwent RAI with lung metastasis+lymph node metastasis. (C) whole body imaging and (D) CT of patients who underwent RAI with bone metastasis. Supplemental Table 1. The baseline characteristics of included participants. [file 12957_2023_3240_MOESM1_ESM.docx]

**
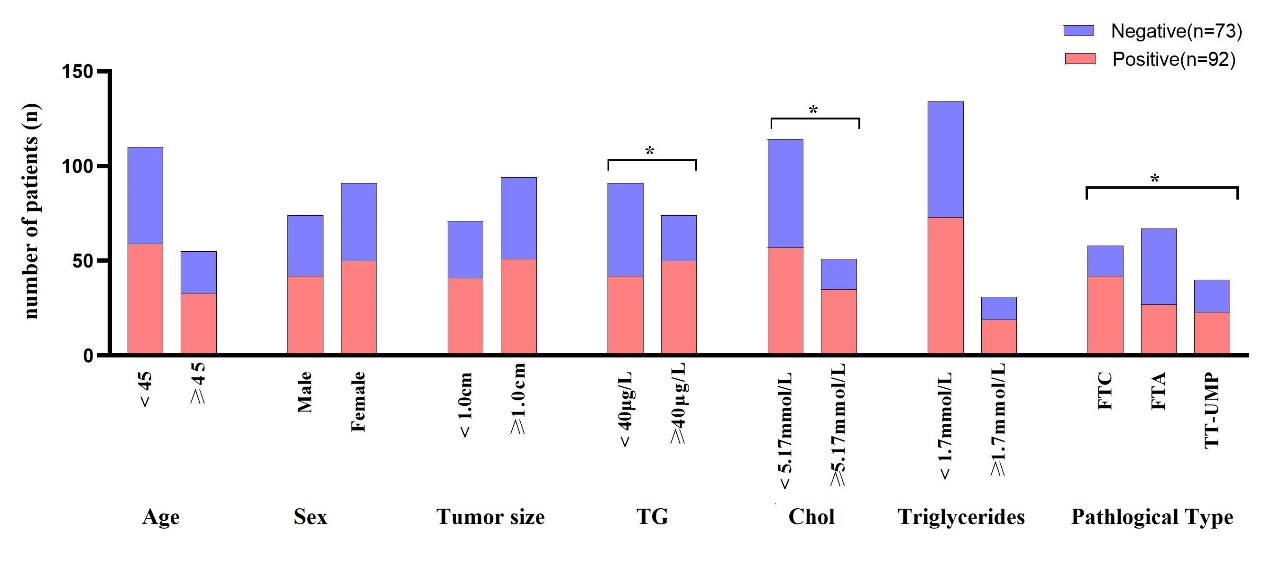
**

**Supplemental Figure 1. Comparison of SLC5A8 gene methylation in patients with different clinical features in baseline**

**
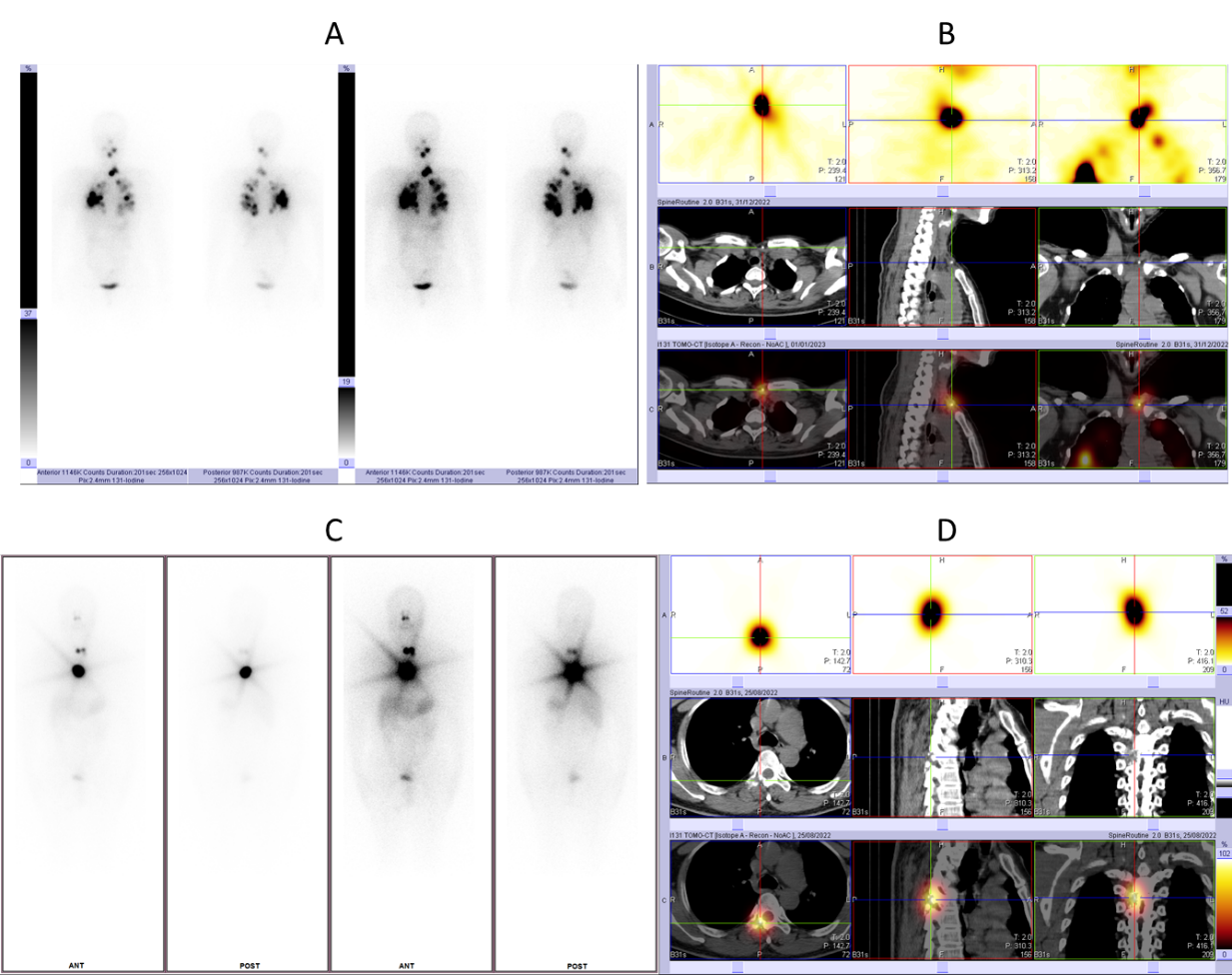
Supplemental Figure 2. Radioactive iodine (RAI) treatment of representative cases with thyroid cancer metastasis.** (A) whole body imaging and (B) computed tomography (CT) of patients who underwent RAI with lung metastasis+lymph node metastasis. (C) whole body imaging and (D) CT of patients who underwent RAI with bone metastasis.

**Lung metastasis+lymph node metastasis**

**Supplemental Table 1. The baseline characteristics of included participants**

| **Variables** | **n** | **Positive(n=42)** | **Negative(n=16)** | **χ^2^** | **P** |
| --- | --- | --- | --- | --- | --- |
| **Histologic type** (n, %) |  |  |  | 1.012 | 0.576 |
| Minimally invasive | 28 | 12(53.6) | 6(46.4) |  |  |
| Angioinvasive | 16 | 11(60.0) | 5(40.0) |  |  |
| Widely invasive | 14 | 9(57.5) | 5 (42.5) |  |  |
