# Supplementary figures and images for: Role of hypermethylated SLC5A8 in follicular thyroid cancer diagnosis and prognosis prediction
Source: World J Surg Oncol. 2023 Nov 25;21:367. doi: 10.1186/s12957-023-03240-1 (PMC10675931; doi:10.1186/s12957-023-03240-1)

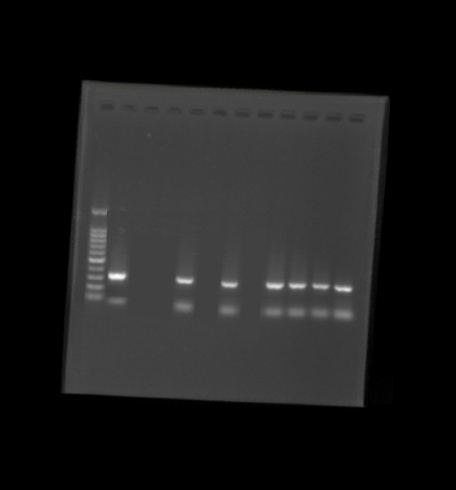

Supplement: Supplementary file 2 — Additional file 2. [file 12957_2023_3240_MOESM2_ESM.zip › Supplemental files of figure 1.jpg]

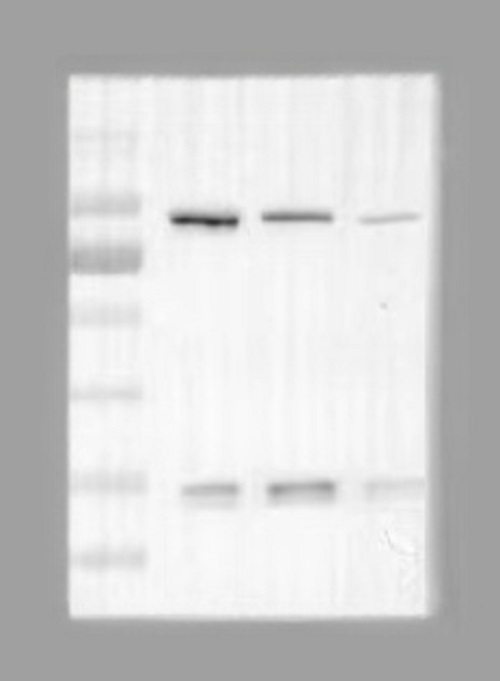

Supplement: Supplementary file 2 — Additional file 2. [file 12957_2023_3240_MOESM2_ESM.zip › Supplemental files of figure 3.jpg]
